# Co-design of a paediatric oncology medicines database (ProCure) to support complex care provision for children with a hard-to-treat cancer

**DOI:** 10.3389/fmed.2024.1332434

**Published:** 2024-03-28

**Authors:** Carolyn G. Mazariego, Skye McKay, Elijah Tyedmers, Lauren Kelada, Brittany C. McGill, Rebecca Daly, Claire E. Wakefield, David S. Ziegler, Natalie Taylor

**Affiliations:** ^1^School of Population Health, UNSW Medicine and Health, UNSW Sydney, Sydney, NSW, Australia; ^2^School of Clinical Medicine, University of New South Wales (UNSW) Sydney, Sydney, NSW, Australia; ^3^Kids Cancer Centre, Sydney Children’s Hospital, Sydney, NSW, Australia; ^4^Children’s Cancer Institute Australia, Lowy Cancer Research Centre, Sydney, NSW, Australia

**Keywords:** paediatric oncology, precision medicine, novel therapies, co-design, implementation science, CFIR

## Abstract

**Objectives:**

Paediatric oncologists often encounter challenges when seeking compassionate access to off-label therapies for their patients. This study employed implementation science and co-design techniques to develop the ProCure medicines database, with the goal of streamlining the application process and addressing identified barriers in paediatric oncology.

**Methods:**

This study utilised an exploratory qualitative research design. Seventeen healthcare providers, including oncologists, nurse consultants, and allied health professionals, participated in semi-structured interviews guided by the Consolidated Framework for Implementation Research (CFIR) and a visual process map aid. Deductive qualitative data analysis, according to the CFIR constructs, identified key barriers and facilitators. Collaborative design sessions engaged multidisciplinary teams to develop the ProCure beta version.

**Results:**

Barriers to off-label therapy access included resource-intensive applications, time sensitive decision-making, and complex pharmaceutical information. Facilitators included Drug Access Navigators, Molecular Tumour Boards, and a multi-disciplinary approach. ProCure addressed end-user needs by centralising medicines information. Additional features suggested by healthcare providers included blood–brain-barrier penetrability data and successful application examples.

**Conclusion:**

ProCure represents a promising solution to the challenges paediatric oncologists face in accessing off-label therapies. By centralising information, it simplifies the application process, aids decision-making, and promotes a collaborative approach to patient care. The potential of the database to stream and enhance off-label therapy access underscores its relevance in improving paediatric oncology practise. Further research and implementation efforts are warranted to assess ProCure’s real-world impact and refine its features based on user feedback.

## 1 Introduction

Precision medicine has ushered in a new era in healthcare, particularly in the field of oncology. It has brought the promise of personalised treatment approaches, underpinned by the identification and validation of biomarkers intricately linked to therapeutic responses (1–3). Central to this paradigm shift is the practise of tumour profiling, a powerful tool that unveils the genetic intricacies indicate cancer specific to the individual, and can reveal potential targeted therapies (3). In this context, precision medicine trials have become a focal point of scientific inquiry and medical practise. These trials have the potential to identify ground-breaking treatment options that hold the promise of improved outcomes for patients. However, a significant challenge looms when these novel therapies, identified through precision medicine, are not approved for use in specific patient populations, most notably in paediatric oncology (4). While there have been significant improvements in treatments for childhood cancer, for an estimated 15% of children diagnosed with cancer, a cure is not possible. For example, Diffuse Midline Gliomas (DMG) represent the most aggressive of all cancers, with almost all children dying within 12 months; we therefore label cancers such as these as ‘hard-to-treat’ (5).

Australia has nine paediatric oncology centres, and it is estimated that ~750 children aged 0–14 years old are diagnosed with cancer each year (6). Given Australia’s vast geographical area, it is estimated that the mean travel distance from home to hospital is >100 km (7).

In Australia, the Therapeutic Goods Administration (TGA) is the regulatory authority responsible for the approval and monitoring of therapeutic goods, including targeted oncology drugs. For paediatric use, the TGA follows a risk-based approach, taking into consideration the specific needs of children (8). Manufacturers are required to provide data on the safety and efficacy of drugs in paediatric populations as part of the regulatory submission. Once approved, they are listed on the Australian Register of Therapeutic Goods (ARTG) for specific indications and populations with appropriate restrictions.

If there are no suitable treatment options available on the ARTG or through clinical trials, a clinician may decide to pursue off-label prescribing based on available evidence. Off-label prescribing means that the TGA has not approved the indication, route of administration or patient group. It does not mean that the TGA has rejected the indication (9). Commonly the TGA has not been asked to evaluate the indication in the context of paediatrics, perhaps due to a lack of robust safety and clinical evidence. For these reasons, prescribing off-label for paediatric patients is often unavoidable. Multiple patients receive off-label therapy at each hospital each year, however, there are no formally reported studies or data available as to how many off-label applications are processed or approved. Anecdotally, seeking off-label drug access is generally more common in children with hard-to-treat cancer.

Furthermore, the Therapeutic Goods Act 1989 (the Act) does not regulate clinical practise. ‘Off-label use’ is a clinical decision, made at the discretion of the treating clinician and does not require an exemption, approval or authorisation from the TGA in order to use a therapeutic good for an off-label use. This clinician is responsible for obtaining informed consent from their patient (which includes telling them if the use is off-label) and ensuring that the agent selected is the most appropriate treatment option.

The TGA may grant special consideration via the Special Access Scheme for compassionate use of imported drugs in situations where there are no satisfactory alternative treatments available in Australia, and the patient has a serious or life-threatening condition. This is generally considered on a case-by-case basis (10).

In contrast, the Food and Drug Administration (FDA) in the United States has specific regulations and guidelines for paediatric drug development. The Paediatric Research Equity Act (PREA) and the Best Pharmaceuticals for Children Act (BPCA) provide incentives for studying drugs in paediatric populations. Paediatric studies may be required for certain drugs, and paediatric labelling information is often included in drug approvals. The FDA also has mechanisms for expanded access or compassionate use, allowing seriously ill patients to access investigational drugs outside of clinical trials. This is regulated under the FDA’s expanded access programme (11).

In the European Union (EU), the European Medicines Agency (EMA) oversees the regulation of medicines. Paediatric requirements are outlined in the Paediatric Regulation, which aims to improve the development and availability of medicines for children. Like the FDA, the EMA encourages the inclusion of paediatric populations in clinical trials and has a system for paediatric investigation plans (PIPs). Compassionate use in the EU is governed by national regulations within each member state. The EMA provides guidance on compassionate use, and the decision to allow such use is made by the relevant national competent authority (12).

The regulatory frameworks in Australia, the FDA, and the EU all prioritise the safety and efficacy of drugs in paediatric populations. Paediatric investigation plans are a common theme, encouraging the inclusion of children in clinical trials. Compassionate use or expanded access programmes exist in all three regions, though specific details and processes may vary.

Regardless of varying practises, paediatric oncologists are at the frontline of dealing with access challenges, as they are the conduit between the patient and pharmaceutical companies, often having to submit lengthy applications to obtain off-label access. Clinicians grapple with a multitude of hurdles, ranging from navigating the regulatory intricacies and negotiating with pharmaceutical companies to meticulously adhering to stringent documentation requirements (4, 13, 14). The absence of readily available resources and comprehensive support exacerbates this already demanding process.

In response to these challenges we developed ProCure, a database which aims to streamline the application process for off-label medicines. ProCure offers a systematic approach designed to expedite the application process, enhance efficiency, promote consistency, and empower paediatric oncologists in their tireless efforts to secure the best possible care for their young patients.

To set the foundation for the design of the ProCure database, this research to explore paediatric healthcare professionals’ (PHCPs): (i) perceived role in identifying and accessing off-label medicines and apply implementation science methods to identify barriers and facilitators encountered, and (ii) PHCPs perceived acceptability of a resource like ProCure and elicit end-user needs to facilitate co-design of the resource.

- Can a database (ProCure) be built to streamline drug information that would be useful for paediatric oncologists when preparing off-label drug access applications?
- What features should ProCure include in order to facilitate ease of use to enable efficiency in the application process for off-label drug access?

## 2 Materials and methods

### 2.1 Participants

This study did not take place in one specific setting (e.g., hospital); we recruited PHCPs from across Australian paediatric clinical sites. Any PHCP (e.g., oncologists, nurses, and pharmacists) who had direct involvement in the care of children or adolescents with hard-to-treat cancers within the previous 24 months, and self-reported experience applying for managing off-label cancer medicines were eligible to participate. In the first instance, known PHCPs were recruited via purposive sampling (i.e., the clinical investigator DZ emailed clinicians involved in Australian paediatric precision medicine trials) and via exponential snowball sampling, with consenting participants opting to provide contact details of other potentially eligible participants. Additional participants were recruited nationally, using study advertisements on social media (i.e., Twitter) and professional network platforms (e.g., Australian and New Zealand Children’s Haematology and Oncology Group). Online REDCap consent was required prior to the interview. This study was approved by the Sydney Children’s Hospital Network Ethics Committee. Informed written consent was obtained from each study participant (HREC Reference: 2021/ETH00583. SCHN HREC).

### 2.2 Data collection

#### 2.2.1 Semi-structured interviews

Individual semi-structured interviews aimed to explore PHCPs’ experiences with off-label therapy applications and access pathways, with a particular focus on challenges and barriers. Following completion of the online consent form the research team (CM, RD, SM, and BM) contacted participants to schedule a suitable interview time.

**Figure 1:**
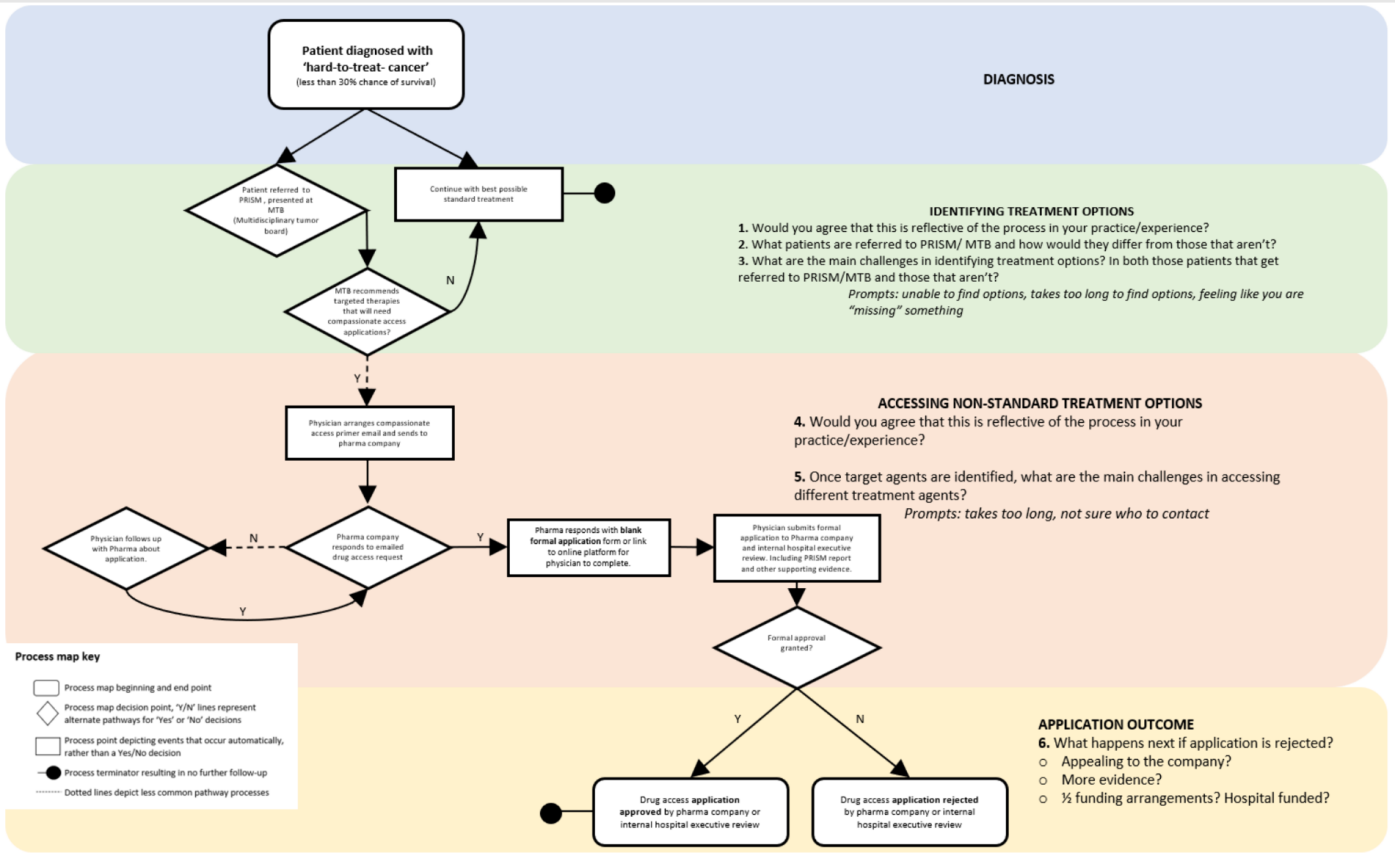
Process map of current off-label access pathway (without ProCure resource support). MTB, Multi-disciplinary Tumour Board; PRISM, refers to the Australian precision medicine trial for children with hard-to-treat cancers; Pharma, Pharmaceutical company.

The first stage of the interview comprised of an implementation process mapping exercise; a visual representation of current practises associated with access to off-label therapeutics in the context of a paediatric oncology precision medicine trial. Implementation process mapping can help to create a visual representation to identify gaps in practise, or how new innovations that facilitate evidence-based practise can be integrated into current workflow (15–17). In order to conduct this process mapping exercise, we began with a draft process map of how the study team understood the steps needed to be taken by paediatric oncologists when attempting to obtain off-label therapies. The interviewer described this process map then asked participants if it was reflective of their experience. We asked participants to confirm or amend these current practises. With each interview, the process map was iterated to reflect amendments and new information (Figure 1). We then asked participants to reflect on the potential usefulness of a resource like ProCure for facilitating/improving current clinical practise.

The second phase of our interview included additional questions guided by the Consolidated Framework for Implementation Research (CFIR) (18, 19). The CFIR to a determinant framework which can aid in systematically identifying factors influencing implementation of interventions at multiple levels within an organisational context. The CFIR comprises a taxonomy containing 31 constructs in five domains as follows: intervention characteristics, outer setting, inner setting, characteristics of the individual, and the process of implementation (18, 19). The CFIR domains and constructs served as a comprehensive framework to guide the interview process and ensure coverage of key potential barriers and enablers to ProCure use and implementation factors. Interviews continued until thematic saturation was reached (defined as three consecutive interviews with no new themes) (20). ProCure was further designed and iterated alongside interview data collection, and where possible, evolving iterations of the database were presented during interviews to obtain feedback.

### 2.3 Data analysis

#### 2.3.1 Qualitative approach

This study utilised an exploratory qualitative research design, employing deductive data analysis (21) through the domains of the widely-used CFIR. Interviews were recorded and then transcribed verbatim using a third-party service. The qualitative interview data were analysed using a deductive thematic analysis approach by coding identified barriers and facilitators to the CFIR.

##### 2.3.1.1 Trustworthiness and rigour

Thirty percent of interviews were double coded by SM and CM (SM is a trained genetic counsellor and CM is an implementation scientist) in NVivo version 12, with any discrepancies resolved through discussion until high intercoder agreement was reached. The coded data were transferred to an Excel spreadsheet and an iterative triangulation approach of developing a coding tree was employed to identify themes and patterns within the data, allowing for the identification of key barriers, end-user needs, and gaps that ProCure should aim to address. SM conducted the rest of the interview coding, and presented themes and extracted illustrative quotes back to the larger study team (CM, ET, and NT) for discussion and finalisation.

#### 2.3.2 Collaborative design sessions

A web-designer (WebWorks) was contracted to help develop an engaging, user-friendly online interface for ProCure. Our implementation science team (CM, ET, SM, and NT) met with the web-designer fortnightly across a 9-month period to design ProCure. Three collaborative design sessions were organised, two with the project steering committee (which consists of researchers, paediatric oncologists, genetic counsellors, and two parents of children with hard-to-treat cancers). The project steering committee was presented the working draft iteration of ProCure and invited to provide informal qualitative feedback (either verbally during the meeting or via email to the study investigators post-meetings) during early/mid-development phases of ProCure. For the third collaborative design session, we arranged for ProCure to be presented to the multidisciplinary team within a precision medicine molecular tumour board (MTB) meeting that comprised of a pharmacist and seven paediatric oncologists. ProCure was presented to the MTB and informal feedback was collected to continue to guide the development of ProCure to be tailor-made for each of use by paediatric oncologists. This session occurred during the late development phase of ProCure (final 3 months). These three collaborative design sessions further facilitated the co-design process, allowing for immediate feedback, suggestions for improvements, and for MTB participants to contribute their expertise to the development of the resource. The feedback from these collaborative sessions was presented back to the web-designer for discussion/exploration of the feasibility of incorporating into ProCure. This manuscript presents the design process for the ProCure resource, however, the study team are also undertaking a formal evaluative Beta testing phase with the ProCure resource that will be reported separately to this design process.

## 3 Results

### 3.1 Participant characteristics

A total of 17 PHCPs participated in the study, comprising nine oncologists and eight clinical nurse consultants/allied health professionals. The average amount of years of experience in delivering paediatric oncological care was 14.5 years (range < 1 year to 41 years, Table 1). This diverse group represented a range of professional roles within paediatric oncology, allowing for comprehensive ‘real world’ insights into the application process for off-label therapies. Interviews took place between June 2021 and May 2022.

**Table 1:** Participant information.

Role	Gender	Years experience
Paediatric Oncologist	F	>20
Clinical Nurse Consultant/Allied Health Professional	F	10–20
Paediatric Oncologist	M	>20
Clinical Nurse Consultant/Allied Health Professional	F	>20
Paediatric Oncologist	F	<5
Clinical Nurse Consultant/Allied Health Professional	F	5–10
Clinical Nurse Consultant/Allied Health Professional	F	10–20
Paediatric Oncologist	F	10–20
Paediatric Oncologist	M	<5
Paediatric Oncologist	M	10–20
Paediatric Oncologist	F	<5
Clinical Nurse Consultant/Allied Health Professional	F	>20
Clinical Nurse Consultant/Allied Health Professional	F	10–20
Clinical Nurse Consultant/Allied Health Professional	F	10–20
Paediatric Oncologist	M	10–20
Paediatric Oncologist	F	5–10
Clinical Nurse Consultant/Allied Health Professional	F	<5

### 3.2 Existing process: identified barriers

The compassionate access application process was identified as resource-intensive, requiring substantial time and effort from PHCPs to identify, compile and submit the necessary documentation (Table 2, with supporting quotes and associated CFIR domains). Barriers included increased PHCP workload related to performing comprehensive literature reviews to support clinical treatment decision-making and identifying the correct pharmaceutical contacts and processes for off-label access applications. The application process was also considered relatively uncommon and unfamiliar to most PHCPs, which was further impeded by the inherent variability between pharmaceutical procedures and complexity of regulatory authority processes (e.g., hospital drug committees, ethics committees). The time-sensitive nature of decision-making regarding off-label therapies was also identified as a significant barrier, placing additional pressure on PHCPs to quickly gather and review relevant pharmaceutical information and to communicate this information to patients and their families.

**Table 2:** Identified barriers and facilitators to off-label therapeutics access.

Barrier (−) or Facilitator (+) theme	Description	CFIR domain	CFIR construct	PHCP quotes
Resource Intensive (−)	PHCP time spent doing a literature review to support each application	Intervention/Inner setting	Complexity/Available resources	[ID_032] ‘The main thing is doing a comprehensive review of the current literature for each diagnosis, and there’s a change in the literature all the time. We’d often go back and re-review the literature for the underlying type of disease the patient has. That would give you a pretty good overview of […] what are regarded to be acceptable therapies, which are likely to be effective, and also what are the newer emerging therapies that have been tried, and also the biology that’s been uncovered in that disease […]. We try and do that for all our patients every time they come back with a hard-to-treat cancer’.
				[ID_063] ‘It depends on the disease, and it depends on what we are looking for, it depends on the drug. […] if it’s one of the drugs that I’ve had on early phase studies and I know on the back of my hand, then it does not take me long, it’s just part of what I do. But if it’s new drug, new disease, something that’s very unique to the patient, that takes a bit longer, and a bit more thought, a bit more literature review, obviously, to supply the evidence’.
				[ID_021] ‘I’d say it’s anywhere from 5 to 20 h [to complete each application]’.
	PHCP time spent identifying correct pharmaceutical contacts and processes	Inner setting	Available resources	[ID_067] ‘If I did not go through [the DAN], then I would have to spend a lot more time in trying to find the correct contact details of the drug companies, because these drug companies are huge and they have different contacts depending on what drug is involved, and trying to find that information can be challenging and then if you get it wrong, you just keep being sent down the line’.
				[ID_014] ‘And [the response from the company] depends on what you say. And not a really extreme process and extremely optimised process, but it depends on who you contact and what do you say when you [make] contact’.
Complex and variable (−)	The off-label application process is an uncommon pathway for PHCPs	Inner setting	Access to knowledge and information	[ID_010] ‘Probably less than ten [applications] a year. The numbers are small compared to the numbers of kids diagnosed. I mean I do not know the answer to that, but it could be between five and ten a year’.
				[ID_014] ‘Maybe six [applications per year] […] it’s a bit tricky, but yes, maybe. And new ones, not intervals. New ones’.
	Variability between pharmaceutical company processes	Outer setting	Cosmopolitanism	[ID_032] ‘If you think about compassionate access, it’s not like there is a standardised way […] in Australia, which all drug companies use. The compassionate access programme is specific to the drug and the drug company. The length of time it might take you to access a particular drug will depend very much on what the process is for that company […] that can be a process of backwards and forwards getting that done’.
	Associated regulatory processes are complicated	Outer setting	External policy and incentives	[ID_009] ‘It may be if you have identified an agent that you believe would benefit the patient, it may be an agent that is currently registered and then the obstacle is getting permission through the hospital to use that agent which may not be freely available or may be very expensive so that there are all the hoops to go through to get permission to use the agent and to pay for it’.
Time sensitive (−)	Pressure to make applications quickly to inform treatment decision-making	Intervention/Process	Complexity/External change agents	[ID_027] ‘We spend enormous amounts of time, particularly when it is time sensitive. You have to send [the application] out within the day in fact or the next day and it’s a lot of work to put in, considering a lot of the other things that are happening in tandem and anything to ease the edge off would be more than welcome’.
				[ID_021] ‘[…] likewise, when I’ve had a review by our hospital drug committee…the information was not provided in a timely fashion, because often there’s a timeline, based on the patient’s condition, that is not necessarily affected by the internal hospital processes, so I’ve had to go back to then the hospital to review where the process was up to and seek confirmation or seek additional documentation as to why certain decisions were made, but that, once again, is quite time consuming’.
The drug access navigator (DAN) (+)	The DAN role provides support to PHCPs during the application process	Intervention	Evidence strength and quality/Relative advantage	[ID_014] ‘I think it’s useful even when we are at the beginning [and] want to explore how feasible is for us to start a drug in a patient that we know that it could be challenging, […] [the DAN] having the knowledge that he has to say, ‘This will most likely be very easy to access because we have precedent and we have all the other cases who they have approved the access to the drug relatively easy.’ And that is already very informative because then you consider [this drug] as the strongest option […]’.
The Molecular tumour board (MTB) (+)	Information about applications can be shared amongst MTB attendees	Inner setting	Networks and communication/Culture	[ID_036] ‘One of the good things about the MTB […] is if there’s someone to have actually accessed the drugs from a company, they will be willing to share that contact – I think people are very nice about it and very helpful. If I’ve applied for a drug with the pharmaceutical company, and then I know at MTB, somebody else is looking for the drug – [I will] generally just volunteer, ‘Here’s the contact I used and this is how you go about it.’ And I think that process with the MTB is very helpful’.
				[ID_021] ‘Part of [which applications have been recently funded or successful] is shared at the MTB level and perhaps that is still the most appropriate’.
Multidisciplinary approach to care (+)	High value placed on multidisciplinary care and consistency in practise	Inner setting	Networks and communication/Culture	[ID_009] ‘There is a safeguard in that most patients are looked after by a group of doctors […] we are screening patients so that different types of cancers are being looked after by people who are specialising in that subgroup of cancers […]. It’s being looked after by people who are keeping abreast of all new developments in that field, which is relatively new […]’.
				[ID_015] ‘I think consistency is the key and we are hoping to become the best centre in southern hemisphere for treating kids with cancer. I think it’s really important for all of the bosses to be on the same page in the way that we are doing things for families in terms of following certain pathways, because when there’s inconsistencies, other families know about it […]. It is really important that we are consistent in what we do in practise’.

Some quotes have been edited to improve readability, without changing the meaning of the text. For example, the removal of filler words (e.g., ‘So’).

### 3.3 Existing process: key facilitators

Paediatric healthcare professionals highlighted the value of a Drug Access Navigator (DAN), who can play a pivotal role in assisting throughout the application process by providing guidance, coordinating documentation, and facilitating communication with regulatory authorities (Table 2). The establishment of a precision medicine Multi-disciplinary Tumour Board (MTB) was seen as another facilitator, enabling multidisciplinary discussions amongst experts to determine the appropriateness of off-label therapies based on molecular profiling and individual patient characteristics, and providing the opportunity to share experiential advice regarding off-label applications. Finally, the adoption of a collaborative, multi-disciplinary and consistent approach to care was emphasised as a facilitator, promoting shared decision-making and comprehensive support for patients.

### 3.4 End-user needs addressed by ProCure

A central aspect of ProCure, which aligns with PCHP needs, is the consolidation of information about medicines within an accessible and well-supported platform. By centralising relevant pharmaceutical information, ProCure was perceived to be a useful and valuable resource (acceptability outcome) that could alleviate the upstream burden on PHCPs in gathering and reviewing scattered data from various sources and potentially standardise drug access applications. One perceived challenge was the capability of maintaining up-to-date literature within a fast-evolving landscape, and some PHCPs expressed the intention to continue to perform their own literature reviews for applications. However, most PHCPs could not identify any major barriers to implementing ProCure into clinical practise.

Paediatric healthcare professionals provided valuable feedback and recommendations to enhance the functionality and acceptability of ProCure, for which high level inclusions and end-user needs are detailed in Table 3. One recurrent suggestion was the inclusion of blood–brain-barrier penetrability data, which would enable PHCPs to make informed decisions regarding the suitability of therapies for central nervous system tumours. PHCPs also expressed the need for examples of successful applications to serve as reference points for application building.

**Table 3:** Recommendations from interviews for inclusions to the ProCure resource.

	Examples of end-user needs/Desires to achieve acceptability	CFIR domain (Construct)	Supporting quotes
The ProCure platform	Well-supported Accessible and user-friendly Simple formatting	Intervention (Adaptability)	[ID_032] ‘I think it’s just easy to find and easy to access is probably the most important thing. I do not need a lot of information […] simple formatting, this is the drug, that’s the pathway’.
	[ID_070] ‘Yeah, that looks like a very, very useful tool […] and it’s very well laid out. [If clinicians] can jump onto something and know how to use it straight away without doing too much training on it, that’s actually a great bonus’.	
ProCure features	Drug profile Drug search and comparison Pharmaceutical contact information Pharmaceutical application criteria Supporting literature and evidence Filter for blood-brain-barrier penetrability A ‘How to Use’ guide	Intervention (Adaptability) (Evidence, strength, and quality)	[ID_047] ‘The things that are listed on the top really—the drug, how it works, the [molecular] targets, scientific evidence to prove its usefulness that would be important, at least as a reference’.
			[ID_036] ‘I think it will be immensely useful to us. I think it will cut a lot of the processes that we have to go through in terms of us trying to figure out who the best person is or what the right contacts are, what the forms are, *etcetera*’.
			[ID_027] ‘I think the crucial question from a drug perspective is for brain cancer patients is, ‘Does it cross the blood–brain barrier?’ I think that’s a primary medical question’.
			[ID_032] ‘[…] having the ability to walk you through the access process would be good because individual levels of comfort […] will depend partly on their own personal experience and [how long] they have been working in the field’.
Potential ProCure features	Examples of successful applications Patient and family friendly section	Intervention (Adaptability)	[ID_021] ‘A suggestion would be if there’s any pre-populated examples of successful applications […] some way of being able to share what has been recently funded or successful’.
			[ID_057] ‘Think [families are] looking for […] evidence-based, trustworthy sites, I think it would be good for them’.

**Figure 2:**
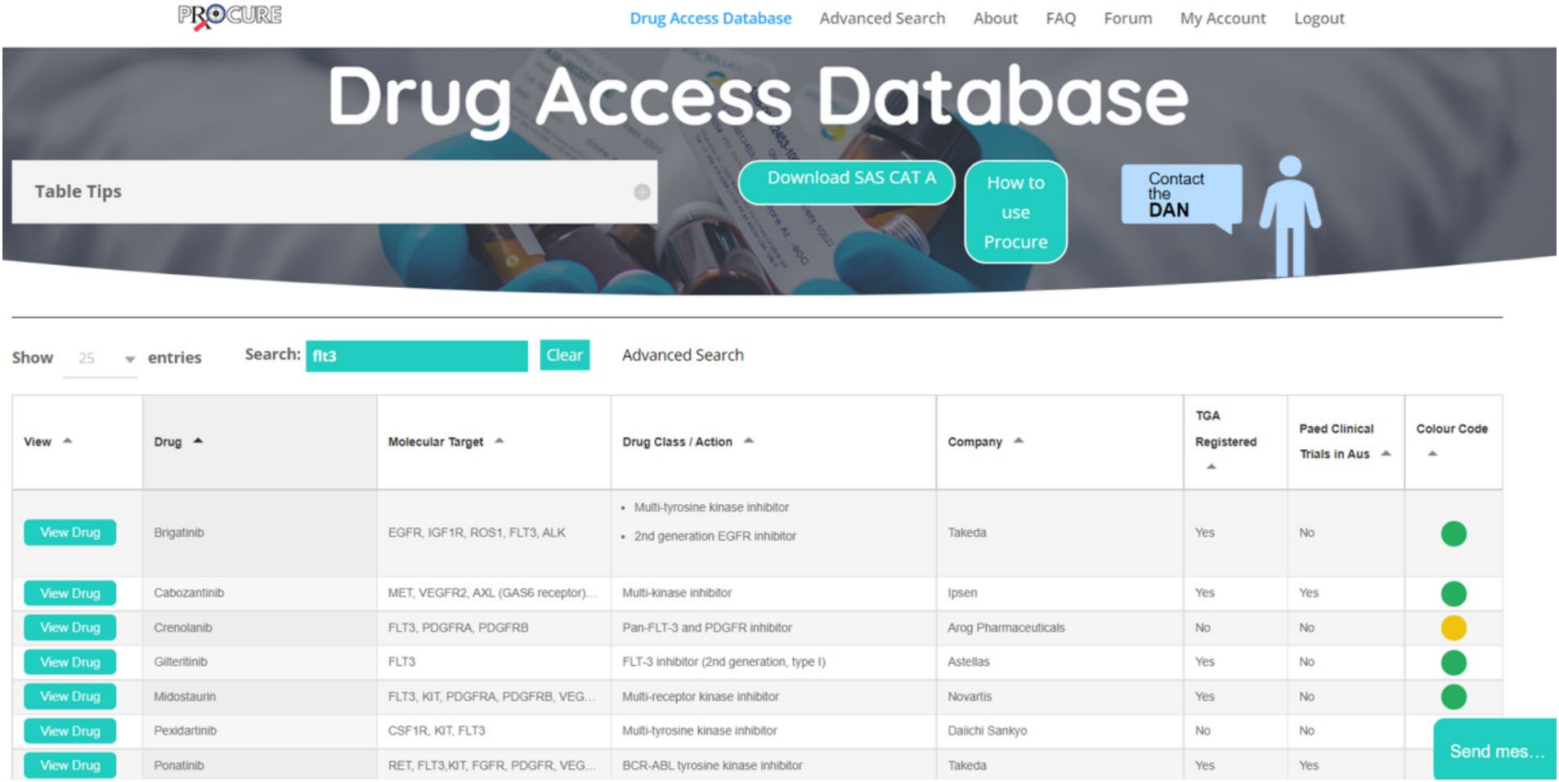
Screenshot of ProCure Beta. Cancer agents and molecular tumour markers can be searched for using the search bar at the top of the database. Searches return relevant results with drug profiles that can be expanded with the button ‘View Drug’. Colour coded dots refer to the level of drug accessibility in Australia; Green, Therapeutic Goods Administration (TGA) approved, established access programme for paediatric indications; a history of compassionate access for certain cancer types and/or molecular targets; known overseas supplier via special access schemes, clinical trial open in Australia; Yellow, Potentially accessible but not TGA approved, may have an access programme for restricted cancers/molecular targets, may have a clinical trial running but no longer recruiting in Australia and may only be recruiting overseas. Compassionate access is possible but less likely; and Red, Appears to be inaccessible to Australia at this time. No approval in any jurisdiction, no open clinical trials in Australia, no access programmes, may have clinical trials open abroad. FAQ, Frequently asked questions; SAS CAT A, Special access scheme—category A (download required when colour code of drug is red); DAN, Drug access navigator; and TGA, Therapeutic goods administration.

Based on the findings from the interviews, identified gaps, and the collaborative design sessions, a beta version of the ProCure database was developed (ProCure-Beta, July 2022; Figure 2).

**Figure 3:**
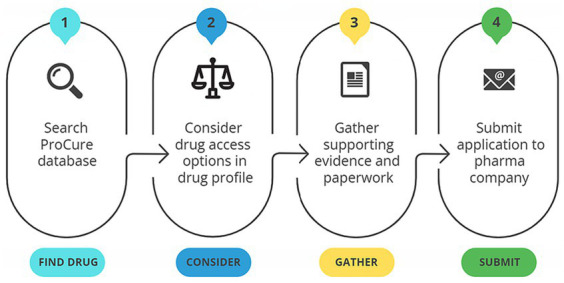
Instructions and steps on how to use the ProCure resource.

ProCure was designed to address the identified barriers, reinforce identified facilitators, and fulfil end-user needs. The study team identified the practical steps through which ProCure would ideally be used (Figure 3), which included: (1) Searching the ProCure resources for the relevant off-label therapy or tumour marker; (2) Consider the drug access options included in the presented search results; and (3) Gather supporting evidence and paperwork (included in the ProCure resource); and (4) Submit application to pharmaceutical contact/company.

## 4 Discussion

This novel study explored PHCPs’ perspectives on their perceived roles in identifying and accessing off-label medicines by applying implementation science methods to identify barriers and facilitators of current processes. The novelty of this study is further strengthened by the end-user centric design of the ProCure database with the outcome of acceptability in mind.

The identified barriers and facilitators provide valuable insights into the challenges currently faced by PHCPs in accessing off-label therapies and the potential impact of ProCure in addressing these challenges. Barriers to off-label therapy access included resource-intensive applications, time sensitive decision-making, and complex pharmaceutical information. Facilitators included DANs, MTBs, and a multi-disciplinary approach. The resource-intensive nature of the current application process aligns with previous research on the complexities and time constraints associated with off-label therapy access (4, 13).

The incorporation of a DAN and the establishment of MTBs as facilitators align with the principles of collaborative and multidisciplinary care, emphasising the importance of interprofessional collaboration in optimising treatment decisions.

### 4.1 Strengths and limitations

Our study employed an end-user centric approach in designing the ProCure resource. This approach increases the likelihood of ProCure aligning with end-user needs and workflow, enhancing its potential for successful implementation (22). However, we acknowledge that the field of precision medicine is rapidly evolving, and so to maintain relevance, ProCure must also evolve to continue to meet end-user needs. This study may not have captured the most recent needs as ProCure was designed in 2022. Ongoing updates and adjustments to ProCure will be required to retain effectiveness and efficiency. Updates to data contained in ProCure are currently facilitated by the DAN, however, for sustainability, we must consider reducing the manual updating aspects of the resource as much as possible, while still maintaining the rigour and relevance in the presented results of performed searches within the resource.

Finally, the use of implementation methodologies and tools to ‘design for implementation’ is a novel approach that this work has undertaken, as implementation outcomes are usually considered once an innovation has proved efficacy (23). The assessment of acceptability as a precursor to practical implementation and ‘real-world’ use of ProCure provided the benefit of being able to identify how to build ProCure to meet end-user needs. In eliciting these end-user needs and building ProCure to match, we established a strong foundation through which we can expect that latter stage implementation outcomes, such as uptake and adoption, would be further realised (19).

### 4.2 Clinical implications

ProCure has the potential to streamline access to off-label therapies for paediatric cancer patients by assisting healthcare professionals in the application and procurement process. By simplifying administrative processes, ProCure can reduce the burden on healthcare professionals, freeing up valuable time and resources, allowing clinicians to focus more on patient care and less on navigating complex paperwork and regulatory hurdles.

Additionally, we envision that ProCure may have secondary effects in enhancing treatment decision making for clinicians. By centralising information about available therapies and molecular profiling, ProCure may empower healthcare professionals to make more informed and targeted treatment choices tailored to individuals. Finally, while ProCure has currently not been designed to serve this purpose, such a resource could potentially be used to not just facilitate off-label access to novel therapeutics, but also to track and monitor off-label use. This could detail data on quality outcomes of off-label therapy use and safety monitoring. These postulations will be explored during the next phase of research, effectiveness and implementation pilot testing.

At present there are no formal evaluations of success of applications to off-label access schemes, however a recent systematic review captured global off-label drug use in adult oncology patients, finding that off-label drug use in inpatients ranged from 18–41% (24). Amongst adult patients with cancer, 13–71% received a minimum of one off-label chemotherapy drug. However, a similar review for the paediatric context is not available, thus we can only speculate how these adult-based data points may be reflective of practises in paediatric care. Ideally, future research would be able to capture some of these data and follow up on instances where we see that access was obtained and how these results might influence policies/regulations within pharmaceutical companies and the ARTG at large.

Finally, ProCure was purpose-built to be iterated, meaning that as new end-user needs are identified they can be functionally added to the resource. Other studies have cited that clear communication between patients, families, and clinicians to manage patient and familial expectations of off-label treatments on a patient’s prognosis is often another challenge clinicians face (13, 25, 26). In future, we may consider a dedicated section of ProCure to provide training or information to clinicians on how best to approach these conversations.

### 4.3 Conclusion

Overall, the findings suggest that ProCure could address the key barriers faced by PHCPs in accessing off-label therapies for paediatric oncology patients. By meeting end-users’ needs for centralised and well-supported pharmaceutical information, ProCure has the potential to streamline the application process, facilitate clinician decision-making, and enhance patient care within the paediatric oncology community.

## Data availability statement

The datasets presented in this article are not readily available because the datasets generated and analysed for this study can be accessed through a separate application to the HREC. Requests to access the datasets should be directed to c.mazariego@unsw.edu.au.

## Ethics statement

The studies involving humans were approved by the Sydney Children’s Hospitals Network Human Research Ethics Committee (SCHN HREC). The studies were conducted in accordance with the local legislation and institutional requirements. The participants provided their written informed consent to participate in this study.

## Author contributions

CM: Data curation, Formal analysis, Investigation, Methodology, Project administration, Writing – original draft, Writing – review & editing. SM: Data curation, Formal analysis, Project administration, Writing – review & editing. ET: Writing – review & editing. LK: Data curation, Writing – review & editing. BM: Data curation, Writing – review & editing. RD: Data curation, Writing – review & editing. CW: Conceptualisation, Funding acquisition, Supervision, Writing – review & editing. DZ: Conceptualisation, Funding acquisition, Supervision, Writing – review & editing. NT: Conceptualisation, Funding acquisition, Methodology, Supervision, Writing – review & editing.

## References

[1] Cahaney C Dhir A Ghosh T. Role of precision medicine in pediatric oncology. Pediatr Ann. (2022) 51:e8–e14. doi: 10.3928/19382359-20211209-01, PMID: 35020508

[2] Hadjadj D Deshmukh S Jabado N. Entering the era of precision medicine in pediatric oncology. Nat Med. (2020) 26:1684–5. doi: 10.1038/s41591-020-1119-6, PMID: 33077957

[3] Lee J Gillam L Visvanathan K Hansford JR McCarthy MC. Clinical utility of precision medicine in pediatric oncology: a systematic review. JCO Precis Oncol. (2021) 5:1088–102. doi: 10.1200/PO.20.00405, PMID: 34994630

[4] Moerdler S Zhang L Gerasimov E Zhu C Wolinsky T Roth M. Physician perspectives on compassionate use in pediatric oncology. Pediatr Blood Cancer. (2019) 66:e27545. doi: 10.1002/pbc.27545, PMID: 30408307

[5] Bartels U Hawkins C Vézina G Kun L Souweidane M Bouffet E. Proceedings of the diffuse intrinsic pontine glioma (DIPG) Toronto think tank: advancing basic and translational research and cooperation in DIPG. J Neuro-Oncol. (2011) 105:119–25. doi: 10.1007/s11060-011-0704-4, PMID: 21901544

[6] Cancer Australia (2022). Statistics for children’s cancers. Available at: https://childrenscancer.canceraustralia.gov.au/about-childrens-cancer/statistics-childrens-cancers

[7] Daniel G Wakefield CE Ryan B Fleming CA Levett N Cohn RJ. Accommodation in pediatric oncology: parental experiences, preferences and unmet needs. Rural Remote Health. (2013) 13:2005. doi: 10.22605/RRH2005, PMID: 23621328

[8] Department of Health and Aged Care (2011). About the work of the TGA—a risk management approach: Australian Government. Available at: https://www.tga.gov.au/about-work-tga-risk-management-approach

[9] Day RO. Ongoing challenges of off-label prescribing. Aust Prescr. (2023) 46:86–9. doi: 10.18773/austprescr.2023.022, PMID: 38152320 PMC10751081

[10] Therapeutic Goods Administration (2023). Special Access Scheme (SAS): Guidance for health practitioners accessing unapproved therapeutic goods. Available at: https://www.tga.gov.au/sites/default/files/2023-02/special-access-scheme-sas-guidance-health-practitioners-accessing-unapproved-therapeutic-goods.pdf

[11] Food and Drug Administration (2016). Drug Research and Children. Available at: https://www.fda.gov/drugs/information-consumers-and-patients-drugs/drug-research-and-children

[12] European Medicines Agency (2024). Needs for paediatric medicines. Available at: https://www.ema.europa.eu/en/human-regulatory-overview/research-and-development/paediatric-medicines-research-and-development/needs-paediatric-medicines

[13] Daly R Hetherington K Hazell E Wadling BR Tyrrell V Tucker KM. Precision medicine is changing the roles of healthcare professionals, scientists, and research staff: learnings from a childhood Cancer precision medicine trial. J Personal Med. (2023) 13:1033. doi: 10.3390/jpm13071033, PMID: 37511646 PMC10381580

[14] Wong M Mayoh C Lau LMS Khuong-Quang D-A Pinese M Kumar A. Whole genome, transcriptome and methylome profiling enhances actionable target discovery in high-risk pediatric cancer. Nat Med. (2020) 26:1742–53. doi: 10.1038/s41591-020-1072-4, PMID: 33020650

[15] Antonacci G Lennox L Barlow J Evans L Reed J. Process mapping in healthcare: a systematic review. BMC Health Serv Res. (2021) 21:342. doi: 10.1186/s12913-021-06254-1, PMID: 33853610 PMC8048073

[16] Kononowech J Landis-Lewis Z Carpenter J Ersek M Hogikyan R Levy C. Visual process maps to support implementation efforts: a case example. Implement Sci Commun. (2020) 1:105. doi: 10.1186/s43058-020-00094-6, PMID: 33292818 PMC7687814

[17] Morrow A Steinberg J Chan P Tiernan G Kennedy E Egoroff N. In person and virtual process mapping experiences to capture and explore variability in clinical practice: application to genetic referral pathways across seven Australian hospital networks. Transl Behav Med. (2023) 13:561–70. doi: 10.1093/tbm/ibad009, PMID: 37036763 PMC10415733

[18] Damschroder LJ Aron DC Keith RE Kirsh SR Alexander JA Lowery JC. Fostering implementation of health services research findings into practice: a consolidated framework for advancing implementation science. Implement Sci. (2009) 4:50. doi: 10.1186/1748-5908-4-50, PMID: 19664226 PMC2736161

[19] Damschroder LJ Reardon CM Opra Widerquist MA Lowery J. Conceptualizing outcomes for use with the consolidated framework for implementation research (CFIR): the CFIR outcomes addendum. Implement Sci. (2022) 17:7. doi: 10.1186/s13012-021-01181-5, PMID: 35065675 PMC8783408

[20] Clarke V Braun V. Thematic analysis. J Posit Psychol. (2017) 12:297–8. doi: 10.1080/17439760.2016.1262613

[21] Azungah T. Qualitative research: deductive and inductive approaches to data analysis. Qual Res J. (2018) 18:383–400. doi: 10.1108/QRJ-D-18-00035

[22] Slattery P Saeri AK Bragge P. Research co-design in health: a rapid overview of reviews. Health Res Policy Syst. (2020) 18:17. doi: 10.1186/s12961-020-0528-9, PMID: 32046728 PMC7014755

[23] Lane-Fall MB Curran GM Beidas RS. Scoping implementation science for the beginner: locating yourself on the “subway line” of translational research. BMC Med Res Methodol. (2019) 19:133. doi: 10.1186/s12874-019-0783-z, PMID: 31253099 PMC6599376

[24] Saiyed MM Ong PS Chew L. Off-label drug use in oncology: a systematic review of literature. J Clin Pharm Ther. (2017) 42:251–8. doi: 10.1111/jcpt.12507, PMID: 28164359

[25] Gereis JM Hetherington K Robertson EG Daly R Donoghoe MW Ziegler DS. Parents' and adolescents' perspectives and understanding of information about childhood cancer precision medicine. Cancer. (2023) 129:3645–55. doi: 10.1002/cncr.34914, PMID: 37376781

[26] McGill BC Wakefield CE Hetherington K Munro LJ Warby M Lau L. “Balancing expectations with actual realities”: conversations with clinicians and scientists in the first year of a high-risk childhood Cancer precision medicine trial. J Personal Med. (2020) 10:9. doi: 10.3390/jpm10010009, PMID: 32075154 PMC7151613

